# Brillouin Spectroscopy: From Biomedical Research to New Generation Pathology Diagnosis

**DOI:** 10.3390/ijms22158055

**Published:** 2021-07-28

**Authors:** Rafael J. Jiménez Rioboó, Nuria Gontán, Daniel Sanderson, Manuel Desco, Maria Victoria Gómez-Gaviro

**Affiliations:** 1 Instituto de Ciencia de Materiales de Madrid (ICMM), Consejo Superior de Investigaciones Científicas (CSIC), C/Sor Juana Inés de la Cruz, 3, 28049 Madrid, Spain; rjimenez@icmm.csic.es; 2 Instituto de Investigación Sanitaria Gregorio Marañón, 28007 Madrid, Spain; 100363421@alumnos.uc3m.es (N.G.); dsanmor09@gmail.com (D.S.); 3 Departamento de Bioingeniería e Ingeniería Aeroespacial, Universidad Carlos III, 28911 Madrid, Spain; 4 Centro de Investigación Biomédica en Red de Salud Mental (CIBERSAM), 28029 Madrid, Spain; 5 Centro Nacional de Investigaciones Cardiovasculares Carlos III, 28029 Madrid, Spain

**Keywords:** Brillouin spectroscopy, diagnosis, mechanics, viscoelasticity, biological tissues

## Abstract

Brillouin spectroscopy has recently gained considerable interest within the biomedical field as an innovative tool to study mechanical properties in biology. The Brillouin effect is based on the inelastic scattering of photons caused by their interaction with thermodynamically driven acoustic modes or phonons and it is highly dependent on the material’s elasticity. Therefore, Brillouin is a contactless, label-free optic approach to elastic and viscoelastic analysis that has enabled unprecedented analysis of ex vivo and in vivo mechanical behavior of several tissues with a micrometric resolution, paving the way to a promising future in clinical diagnosis. Here, we comprehensively review the different studies of this fast-moving field that have been performed up to date to provide a quick guide of the current literature. In addition, we offer a general view of Brillouin’s biomedical potential to encourage its further development to reach its implementation as a feasible, cost-effective pathology diagnostic tool.

## 1. Introduction

As the role of mechanical properties of cells and tissues is gaining high relevance in the study of a wide range of biological processes, Brillouin imaging has emerged as a promising tool for the characterization of biological samples in terms of their viscoelastic behavior. Traditional techniques for the characterization of biomaterials such as magnetic bead twisting, deformation microscopy, micro-rheology, or atomic force microscopy (AFM) either require contact, are destructive, or do not provide sufficient resolution. Conventional optical coherence elastography, which is a clinical tool that measures tissue biomechanics, is very sensitive to environmental vibrations [1]. On the contrary, Brillouin imaging presents a contactless, label-free, non-destructive modality for probing biological samples in the GHz/micron scale and with great potential in clinical diagnosis. 

The Brillouin light scattering (BS) effect was predicted by Léon Brillouin [2,3] and Leonid I. Mandelstam [4,5,6] independently in 1922 and 1926, respectively. However, there is controversy due to the possibility that L. I. Mandelstam had already presented this effect in 1918 [7]. L. I. Mandelstam joined Eugenii Gross to detect the BS experimentally, becoming the first person to observe the Brillouin effect and offer empirical confirmation [8]. 

The laser’s invention in the 1960s brought a light source powerful enough to reduce acquisition times and increase the resolution, resulting in hundreds of experimental works in the area of condensed matter and becoming a consolidated tool [8,9]. Following the theoretical model presented by L. Brillouin and L. I. Mandelstam, Brillouin–Mandelstam scattering (for the sake of simplicity, it will be only denoted as Brillouin scattering (BS)) is based on the light’s interaction with collective fluctuations of density in the physical medium, provoking a change in the frequency of the scattered light; this effect is explained by a sort of Doppler effect in addition to the Bragg condition to obtain constructive interference. 

**Figure 1:**
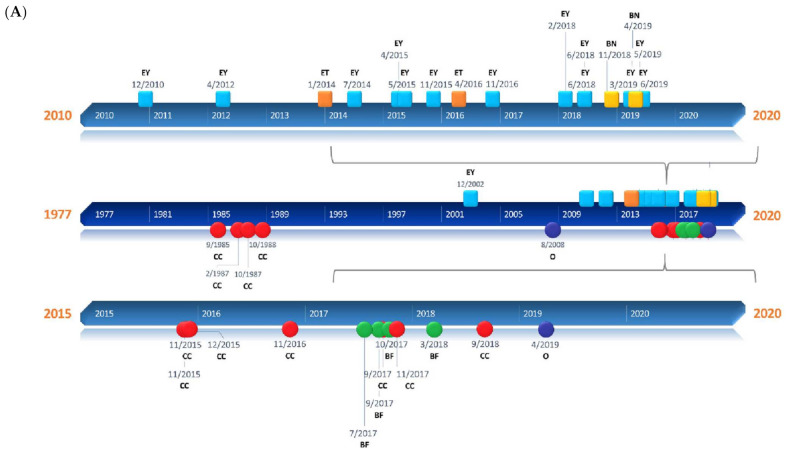

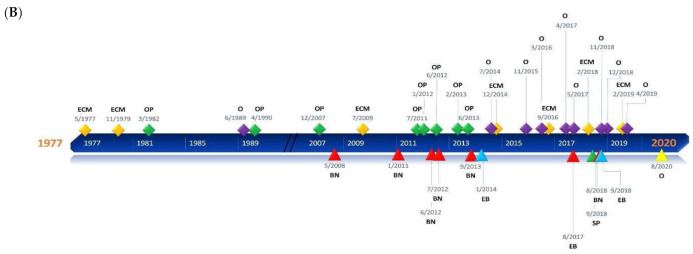
Timelines of the different applications presented in the review. (**A**) Square milestones represent publications related to human applications of Brillouin and have been classified into three groups: ophthalmology (EY), epithelial tissue (ET), and bone (BN). Circular milestones represent publications related to Brillouin microscopic studies regarding subcellular components (CC), biofilms (BF), and others (O). (**B**) Rhomboidal milestones represent Brillouin studies of animal tissues such as extracellular matrix (ECM), ephthalmological tissues (OP) such as the cornea or crystalline lenses, and others (O) that include neuronal, vascular, and oncological applications, among others. Triangular milestones represent publications related to Brillouin studies of animal organs such as bone (BN), spine (SP), embryos (EB), or others (O).

The first examples of BS measurements on biological tissues were described in the late 1970s and early 1980s that demonstrated the power of this system as a research tool of high resolution and sensitivity [10,11]. Following the development of the virtually imaged phased array (VIPA) spectrometers in 1996 and the introduction of Brillouin imaging in 2005, the topic has flourished into a prolific and fast-moving research field [12,13,14]. During the last decade, a great variety of applications of BS to biology and biomedicine has been reported. BS has been tested as a screening and diagnostic tool and a monitoring resource for a wide range of biological samples at cellular and tissue resolution [15]. This work aims to offer an overview of the development of BS-based techniques in biology, offering a comprehensive summary of the currently available literature and state-of-the-art instrumentation, and explores possible future outlooks (Figure 1A,B).

## 2. Brillouin Light Scattering

### 2.1. Physical Basis 

Scattering of light results from the interaction of the electromagnetic field with the constituents of the physical medium. That is the reason why a laser beam can be seen in directions other than the propagation direction. The scattered light can be more or less intense depending on the density of the physical medium and the particle size. The scattered light is composed of different components: one component corresponds to the original frequency (Rayleigh) and others result from intense frequency changes (Raman) or very subtle ones (Brillouin). Apart from the energy change in the scattered light, another relevant difference between Raman and Brillouin scattering includes the physical origin of the effect. In a simplified manner, the intramolecular vibrations are relevant in Raman scattering and the low energy collective vibrations are relevant for Brillouin scattering. The magnitude of the Brillouin-scattered light is about 10^3^ times smaller than the Raman component and its frequency changes only about 10^−6^–10^−5^ times with respect to the incident laser light; therefore, it is very challenging to assess. 

The classical explanation of the Brillouin effect relies on the interaction of the electric field of the light with thermal fluctuations of the dielectric constant of the physical medium as first described in 1880 by the Lorentz–Lorenz equation. A historical review of this formula is presented by H. Kragh [16]:

(1) $$\frac{n^{2}-1}{n^{2}+2}=r\;\rho$$

where *n*^2^ = *ε*; *n* is the refractive index and *ε* is the dielectric constant, *r* is the specific refractivity, and *ρ* the mass density. 

**Figure 2:**
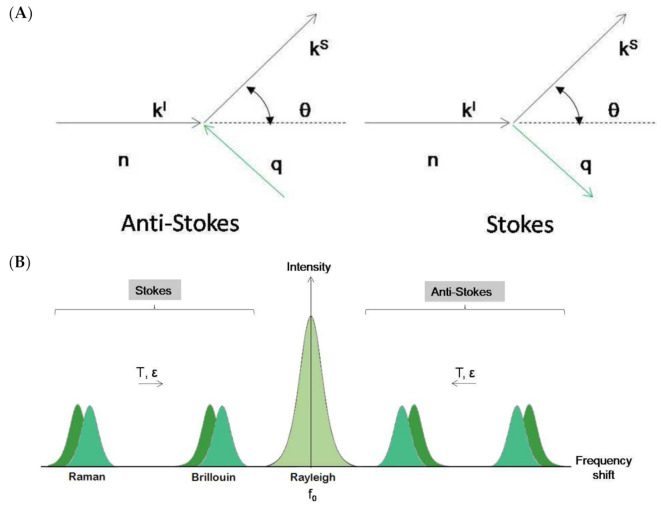
(**A**). Schematic representation of the Brillouin–Mandelstam effect as a collision between photon (***k^l^***) and phonon (***q***). n is the refractive index of the physical medium and *θ* is the scattering angle. (**B**). Schematic representation of Brillouin and Raman spectra. In both experimental techniques, a small amount of light undergoes inelastic scatter, gaining or losing energy and giving rise to a couple of symmetric, frequency-shifted peaks in the spectrum. The remaining elastically scattered light (Rayleigh scatter) appears as a broad central peak with the same frequency as the incident light (*f*_0_). In Brillouin and Raman spectroscopies, peak frequency position varies with temperature (*T*) and strain (*ε*). In Raman spectroscopy, the intensity ratio between Stokes and anti-Stokes peaks varies also with *T*.

However, a new vision of the Brillouin–Mandelsatm effect arises with the introduction of quantum mechanical approaches. Light is constituted by photons that behave as if they suffer an inelastic collision when traversing a physical medium (Figure 2A). The counterpart of this collision is the collective vibrations of the medium. These vibrations can be expressed in eigenmodes and can thus be treated as quasiparticles (phonons). In an inelastic collision, energy and momentum must be conserved, resulting in the following relationships:

***k****^s^* − ***k****^i^* = ±***q*** (momentum conservation) ω*^s^* − ω*^i^* = ±ω(***q***) (energy conservation) (2)

where ***k****^s^* and ***k****^i^* correspond to the scattered and incident light wave vectors, ***q*** is the acoustic wave vector, ω*^s^* and ω*^i^* correspond to the scattered and incident light’s physical frequencies, and ω(***q***) is the acoustic frequency. The shift in the probing light’s frequency is therefore equivalent to the acoustic frequency of the phonons and it is known as the Brillouin shift. Hard media have high acoustic frequencies and therefore larger Brillouin shifts than soft media. The Brillouin shift is also highly dependent on the temperature of the media as thermal energy increases the vibrational frequency of phonons (Figure 2B). 

This picture, which may be mathematically but not conceptually simple (Figure 2A), is well established in solid state physics and is based on a crystalline lattice. In systems without crystalline lattice, as is the case of liquids or glasses, the concept of hydrodynamic modes has been successfully introduced [17,18]. A typical Brillouin scattering spectrum is shown in Figure 2B. 

The stimulated Brillouin spectroscopy (SBS) is a conceptually different method that has been recovered and is being considered for biomedical research. This method has shown its potential in liquids and some polymers [19], and is based on the interference of two incident laser beams traversing the sample in different directions but crossing in a defined sample region. A density diffraction grid is stimulated (mainly by electrostriction) and can propagate in the physical medium. Its propagation velocity corresponds to an acoustic wave’s propagation velocity and is detected by one of the lasers. SBS imaging enables measurements with fast acquisition times free from elastic scattering background; therefore, it has been presented as a compelling alternative to conventional BS [20]. However, as SBS is based on the simultaneous application of two lasers, internal scattering may entail a critical hindering in its applicability to non-transparent samples if an initial optical clearing step is not performed. Nevertheless, its Raman homologous SRS has been successfully applied to non-cleared 1 mm thick mouse brain sections with a cellular resolution [21]. 

There is a growing tendency to combine Brillouin with Raman spectroscopy; therefore, a brief insight on the physical basis of the Raman effect should be provided to understand its contribution to some of the Brillouin studies that will be presented in this review. Both Brillouin and Raman scattering are based on the principle of inelastic scattering of light by phonons that are acoustic in the case of Brillouin and optical in the case of Raman. While the probing of acoustic phonons by BS provides information about the speed of propagation of lattice vibrations, which relates to the mechanical properties and density of the material, the probing of optical phonons by Raman yields information concerning the intrinsic vibrations of molecules, indicating the types of bonds that exist between them and allowing for the molecular characterization of samples. More in depth, Raman scattering is caused by intermediate electronic states that arise due to quantum vibrations specific for different molecular bond types, leading to asymmetric electronic transitions that vary in their final resting state. Raman is not a type of fluorescence emission that is characterized by energy loss in the excited electronic state but for which the resting state corresponds to the pre-excitation one [22]. Contrarily, in Raman, the electrons do not suffer energy loss in the excited state but relax into a different resting-state depending on the corresponding bond’s vibrational energy, inducing a Raman frequency shift proportional to such a vibrational state [22].

### 2.2. Experimental Setup: Scattering Geometries

The experimental implementation of a Brillouin spectroscopy system is somewhat complicated as the photon–phonon interaction occurs within the material under study and must be described in a reference frame outside the material, namely the laboratory reference frame. This fact is very relevant and therefore the so-called scattering geometries is crucial in the BS experiment. The scattering geometries are geometrical relationships between the sample orientation, incident, and scattered light beams in the laboratory reference frame. Moreover, these scattering geometries determine the direction and magnitude of the acoustic scattering wave vector (***q***) that is fundamental for the correct interpretation of the BS experiment in general and particularly in the case of material with preferential directions as in crystals or fibers.

**Figure 3:**
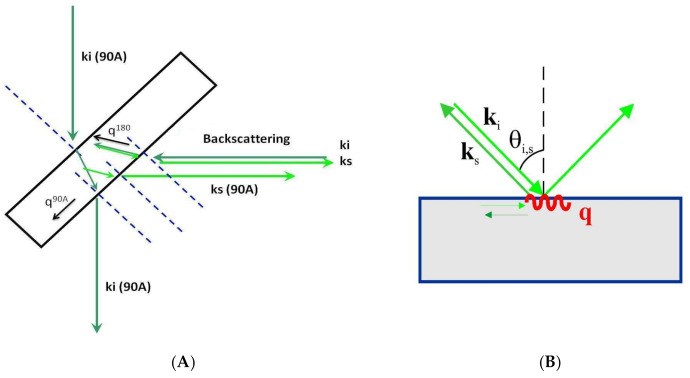

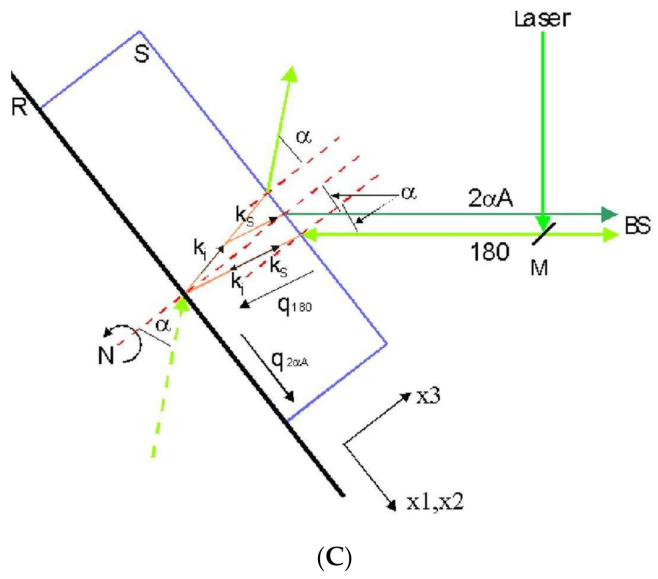
Representation of different scattering geometries. (**A**) 90*A* and 180 as used in solid transparent samples or liquids inside a cuvette. ***k****_i_* and ***k****_s_* are incident and scattered optical wave vectors, respectively. ***q***^90*A*^ and ***q***^180^ correspond to acoustic wave vectors. (**B**) Scattering geometry in an opaque material. (**C**) Scattering geometries in a film on a reflecting substrate. The reflected light beam acts as a virtual one from behind the sample. Two scattering geometries exist simultaneously: 180 and 2*αA*.

The most commonly used scattering geometries are 90, 90*A*, and 180 (backscattering geometry) [23]. Figure 3A shows the 90*A* and 180 (backscattering geometries) with the incident’s geometrical relations, scattered light directions, and the acoustic wave vectors involved. As can be observed, different scattering geometries imply different orientations of the acoustic wave vector. The magnitude of the acoustic wave vector is provided by:

(3) $$q^{90A}=\frac{2\pi \sqrt{2}}{\lambda_{0}}\;q^{180}=\frac{n4\pi }{\lambda_{0}}$$

where *λ*_0_ is the laser vacuum wavelength and *n* is the refractive index of the physical medium. The wave vector’s magnitude in the 90*A* scattering geometry is independent of the refractive index of the material studied. In contrast, in the other scattering geometries, the refractive index is always present. The combination of these scattering geometries in an optically and elastically isotropic material provides the value of the corresponding refractive index:

(4) $$n=\frac{f^{180}}{f^{90A}\sqrt{2}}$$

where *f*^90*A*^ and *f*^180^ correspond to the Brillouin frequency shift of the experiment for the 90*A* and backscattering geometries, respectively. 

A compelling case concerns transparent film samples (thickness above 500 nm), either self-standing or resting on a reflecting substrate. As can be observed in Figure 3C, on the back surface of the plate, the incident light reflects and produces a supplementary light source that allows the simultaneous observation of two scattering geometries including the 180 (backscattering) and the other depending on the incident angle [24]. This second scattering geometry can be denoted as 2*αA*, similar to the 90*A* because its acoustic wave vector is independent of the refractive film index:

(5) $$q^{2\alpha A}=\frac{4\pi \sin\alpha_{i}}{\lambda_{0}}$$

where *α_i_* is the incident angle. 

In opaque materials with an excellent surface optical quality of mostly metallic or semiconductor materials, the light beam is reflected on the surface and cannot penetrate the inner part of the sample. Only the projection on the material’s surface of the incident and scattered light wave vectors are relevant for the scattering process. The acoustic wave vector remains on the material’s surface and can only couple to its surface acoustic waves (SAW) (Figure 3B). In this case, the acoustic wave vector reads as follows [25]:

(6) $$q^{SAW}=\frac{4\pi \sin\theta_{i}}{\lambda_{0}}$$

where *θ_i_* is the incident angle. In magnetic materials, the magnetic field of the incident light can couple to the magnetic modes of the material (magnons) to obtain information about its magnetic properties [26].

### 2.3. Mechano–Elastic Information of the Brillouin Signal

**Figure 4:**
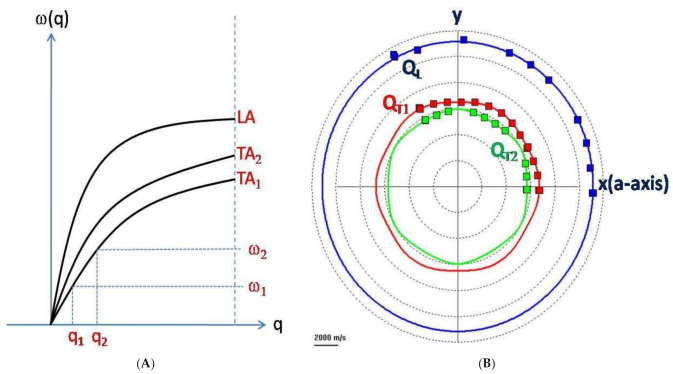
(**A**). Principle of dispersion curves for acoustic phonons of a crystalline material. There are three branches of acoustic phonons: one longitudinal (LA) branch and two transverse (TA_1_ and TA_2_) branches. A change in the acoustic wave vector (***q***) implies a change in the Brillouin frequency shift (*ω*). This indicates that the geometry of acquisition must be carefully assessed as different acoustic modes can fall on the same frequency shift for different geometries. (**B**). Orientation dependence of the sound velocity of sapphire in the (0001) plane (own data). *Q_L_* is the quasilongitudinal acoustic branch, while *Q_T_*_1_ and *Q_T_*_2_ are the two quasitransverse acoustic branches. x and y represent the main crystallographic directions. *Q_T_*_1_ and *Q_T_*_2_ are present because sapphire is an anisotropic crystal and as such, it has lateral and top-down transversal polarizations. These modes depend on the orientation of the crystal. In this case, each angular point of the color curves of the diagram represent Brillouin measurements acquired for different rotations of the x–y plane around the z axis (perpendicular to the plane). Preferential directions along which sound waves can propagate more easily appear as larger shifts of the transverse modes.

The position of the Brillouin peaks in the spectrum is related to the change in frequency (energy) due to the BS effect (Figure 2B). This change is a characteristic of the material under study and depends on the magnitude and direction of the selected acoustic wave vector. This dependence is known as acoustic dispersion (Figure 4A). There are three acoustic modes of the crystalline lattice propagating in the same direction in a crystalline material. One is the longitudinal mode and the other two are the transverse modes [27,28]. An important note is that the notion of acoustic modes refers to the acoustic polarization and not to the direction of propagation. The longitudinal mode corresponds to the cyclic compression–expansion of the media within a phonon in the propagation direction of the acoustic vector, similarly to any sound wave. The transverse modes correspond to shear displacements of the media that occur perpendicularly to the propagation direction. Shear displacements are energetically weaker and thus more difficult to detect than longitudinal modes. In addition, transverse modes do not always result in opto-elastic coupling and therefore may not be reflected in the BLS spectrum. 

If the propagation direction of the acoustic modes coincides with one of the main crystallographic axes, these modes are directly related to the elastic constants of the material along the axis. As an example of the possibilities of the BS as applied to anisotropic samples, Figure 4B shows the orientation dependence of sapphire’s three different acoustic modes in the (0001) crystallographic plane obtained in 90*A* scattering geometry. As explained above, in the BS experiment, the acoustic wave vector’s magnitude and direction are fixed in the laboratory reference frame. Thus, it is possible to choose the direction of the acoustic wave vector and couple the Brillouin modes to different elastic constants of the material. In an isotropic elastic system where all space directions are equivalent, the BS spectrum has only two components: the acoustic longitudinal mode that is related to the longitudinal elastic constant c_11_ and the transverse mode that results from the degeneration of the two transverse modes into a single one that reflects the shear elastic constant c_44_. In liquid samples, only one acoustic mode is expected and corresponds to the elastic constant c_11_ or adiabatic bulk modulus *K_s_*. 

In principle, the relationship between the elastic constant and the Brillouin frequency shift is provided by:

(7) $$c=\rho \frac{\omega^{2}}{{\left|q\right|}^{2}}$$

which in the case of backscattering geometry (the most common case in biomedical applications) results as:

(8) $$c=\rho {\left(\frac{f^{180}\lambda_{0}}{2n}\right)}^{2}$$

In an amorphous or isotropized solid material, the relationships between the longitudinal and shear elastic constants *c*_11_ and *c*_44_ and the typical mechanical constants are the following [29,30]:

*c*_12_ = *c*_11_ − 2*c*_44_
Shear modulus: *μ*, *G* = *c*_44_
Young’s modulus: *Y*, *E* = *c*_44_ (3*c*_11_ − 4*c*_44_)/(*c*_11_ − *c*_44_)
Poisson’s number: *ν* = (*c*_11_ − 2*c*_44_)/(2*c*_11_ − 2*c*_44_)
Adiabatic Bulk Modulus: $K_{s}=c_{11}-\frac{4}{3}c_{44}$.

In biological samples, these mechanical constants generally depend on a few more elastic constants. In several cases, they cannot be computed because the intense background noise and broad Rayleigh peak obliterate the transversal modes and make it impossible to obtain the shear elastic constants. Therefore, only the longitudinal elastic moduli may be obtained, which represent the 1D elastic constant of the Hook’s law, in contraposition to the Young’s Modulus that represents the same physical phenomena but in 3D.

When addressing liquids and viscoelastic materials, the elastic constants become a complex number (c_kl_* = *c_kl_*′ + *i c_kl_*″) in which the real part (*c_ij_*′) corresponds to what has been discussed above and the imaginary part (*c_kl_*″) depends on the frequency and the longitudinal viscosity of the material (*c_kl_*″ = *ω^B^ηL*). The longitudinal viscosity is reflected in the width of the Brillouin peak via:

(9) $$\Gamma^{B}=\frac{1}{2}\rho \eta_{L}q^{2}$$

*Γ^B^* is the half width at the half maximum point of the Brillouin peak [18]. Alternative formulations relate *Γ^B^* to the hyper-acoustic attenuation within the sample [31]. Unfortunately, *Γ^B^* is much more affected by external conditions (i.e., optical quality of the sample surface among others) than *f^B^* that modifies the magnitude of the width of the Brillouin peak and renders very difficult to obtain precise statements regarding the actual physical processes behind *Γ^B^* (see Section 2.4). 

When applying Brillouin spectroscopy to biomedical research, it is important to consider the influence of the water content of the sample on the Brillouin shift. For highly hydrated tissues, the Brillouin shift is mostly provided by water’s compressibility, rendering it very sensitive to water content [32,33]. The reason for this is that organic samples can be modeled as biphasic with a solid and liquid component, each one contributing to the net longitudinal modulus as provided by the following relationship [33]:

(10) $$\frac{1}{M}=\frac{\epsilon }{M_{l}}+\frac{1-\epsilon }{M_{s}}$$

where *M* corresponds to the overall longitudinal modulus, *ϵ* represents the fraction of water content in the sample, and *M_l_* and *M_S_* correspond to the sample’s liquid and solid longitudinal moduli phases, respectively. The above equation’s physical meaning is straightforward: from an elastic perspective, biphasic systems such as living tissues can be modelled as two springs placed in series. The concentration of each one of the phases is the length of the spring. The stiffer and the shorter the spring is, the less it contributes to the overall stretching. Solid materials have a much larger longitudinal modulus than liquids; that is, they are stiffer. Therefore, for highly hydrated samples, the Brillouin shift is mainly provided by the amount of hydration and the water’s compressibility.

### 2.4. Instrumentation

The Brillouin frequency change in the light beam associated with its interaction with density waves is minimal (about 10^−6^). In addition, the correct quantity of the photons related to this effect is small. Compared to a typical Raman spectroscopy experiment, the amount of BS-related photons is about three orders of magnitude smaller than the standard Raman photon quantity. A new kind of spectrometer and a powerful monochromatic light source was required to improve this new light scattering technique. The latter was obtained with the LASER invention and the former was achieved using the constructive interference phenomenon of light in an optical system of the Fabry–Pérot type. The final qualitative leap was introduced by J. Sandercock and his tandem Fabry–Pérot-based spectrometer [34]. The available spectral range was enlarged and the contrast enhanced due to the suppression of the higher interference orders in the spectrum. Apart from the better quality of the recorded spectra, this breakthrough allowed the BS to enter new applications to study SAW and magnons. However, this experimental technique suffered from long acquisition times for each spectrum. This last point is essential and it is why this technique was not widely applied to the biology field until recent years. In biology, fast acquisition times are required to provide maps of the elastic behavior variations in biological samples. This drawback of the Sandercock’s system was overcome by the introduction of a new spectrometer not based on the principle of the standard Fabry–Pérot but on a virtually imaged phase array (VIPA) and the use of CCD cameras [35,36]. The acquisition times were reduced by several orders of magnitude down to Raman spectroscopy’s typical acquisition times [37]. Technical improvements of VIPA spectrometers have continued to enhance performances and simplify the optical setup within the spectrometer [38].

However, there is controversy when trying to determine which spectrometer is adequate for specific applications. In the recent years, Sandercock’s system has been improved and now it is also able to perform Raman and Brillouin measurements simultaneously [22]. These developments are the result of the application of the BS to biomedical challenges. While the development of the VIPA spectrometer is a result of biomedical research, the standard Fabry–Pérot spectrometer is now more versatile. It allows for its use in different scientific fields by forming slight modifications in the optical path to use micro-Brillouin or conventional BS but at the cost of increased acquisition times. 

These latest developments on the spectrometers’ performances, although impressive, are not enough to achieve the goal of BS clinical applications in situ. To understand this point, it is necessary to find a different way to send the light to the sample and then collect it that is not dependent on microscope objectives. A promising way involves the proposition of the use of optical fibers to send and collect the Brillouin scattered light [39]. There are some technical problems in using optical fibers but they can be overcome at least partially, allowing for the obtainment of reliable measurements in simple liquids. In fact, a fiber optic Brillouin probe has been recently developed that is free of background signals and has state-of-the-art spectral resolution [40]. It is the first step to a “Brillouin endoscope”. 

As of 2008, a common approach in biomedical research has been to combine a Brillouin spectrometer with an optical or confocal microscope as a tool to focus the incident laser beam and collect the scattered light [35]. In this manner, complete images can be acquired by scanning the sample to characterize its mechano–elastic properties pixel by pixel. 

However, the use of a microscope limits the experimental setup to a backscattering geometry because only the scattered light that follows the same optical path as the incident beam is collected. The backscattering geometry forbids the coupling of the incident light beam to the transverse acoustic modes; its elasto-optic coupling is zero [41]. Therefore, the use of a microscope implies that only the longitudinal elastic constants of a sample can be obtained.

#### Influence of the Testing Frequency

In the Brillouin–Mandelstam experiment, the acoustic wave vector’s direction and magnitude are fixed in the laboratory reference frame. The obtained spectrum presents peaks at frequencies of several GHz that are significantly high, especially in materials showing acoustic dispersion. Depending on the frequency testing the sample material, the obtained elastic information may be puzzling or incoherent compared to other experimental techniques. This fact was already noticed in some materials when comparing ultrasonic data with Brillouin data (MHz vs. GHz). Even mechano–elastic effects present in the Brillouin experiments become invisible in the ultrasonic ones [42]. In viscoelastic media, these disagreements become more pronounced. Usually, experimental techniques used to characterize the elastic behavior in viscoelastic materials are based on micro-nanoindentation or AFM techniques that use testing frequencies of about some Hz or even work in quasistatic mode. Therefore, it is not astonishing to observe significant discrepancies between the experimental data obtained with those techniques and the experimental data obtained by BS that uses a testing frequency of about some GHz. This can be better understood if we consider water as an example. Water is a Newtonian fluid whose viscous stress is linearly correlated to the rate of deformation produced by an external agent. This indicates that the harder/faster an object attempts to hit/deform water, the more rigid the response of water will be. Therefore, any attempt to correlate elastic characterization results obtained by BS with the results obtained by micro-nanoindentation and AFM in biological systems will be sterile and will not lead to general conclusions. 

The same experiments performed on solid materials such as a window glass or crystals demonstrate no differences between different experimental techniques, providing no acoustic dispersion. For instance, in the data sheet of Schott D263M Glass Coverslips, the value of the Young’s modulus is of 72.9 kN/mm^2^ (https://www.tedpella.com/histo_html/coverslip-info.htm, accessed on 1 July 2021); in the case of the coverslips from the Präzision Glass and Optik (https://www.pgo-online.com/intl/0211.html, accessed on 1 July 2021), the Young’s modulus is of 7.59 × 10^3^ kg/mm^2^, and from the Brillouin experiment values on a similar cover glass, the obtained value is of 74 GPa [43].

### 2.5. Brillouin Scattering and Optical Tissue Clearing 

There are some inconveniences when addressing non-transparent biological samples. Brillouin signal acquisition is limited by laser penetration in non-transparent samples and by undesired light scattering within them [33]. Additionally, refractive index variations throughout the sample account for 2–3% of Brillouin shift variability in backscattering geometry acquisitions, limiting the resolution of the computed longitudinal elastic modulus [22,44]. In 2019, to overcome these issues and improve Brillouin detection, we applied CUBIC optical clearing to murine heart and brain sections. CUBIC consists of two steps: lipid removal by CUBIC R1 and refractive index (RI) homogenization by CUBIC R2. We obtained an improved Brillouin signal with increased peak intensities, following results obtained some years earlier [45,46,47]. This is due to an increase in the number of photons returned to the microscope by the sample due to a reduction in internal scattering. However, this can potentially affect the performance of dispersive spectrometers due to an increase in the Rayleigh central peak (*I_R_*) proportional to the sum of Brillouin peaks (2*I_B_*) as expressed by the Landau–Placzek ratio [32]:

(11) $$\frac{I_{R}}{2I_{B}}=\frac{\beta_{T}-\beta_{S}}{\beta_{S}}$$

where *β_S_* and *β_T_* correspond to the adiabatic and isothermal compressibilities, respectively. Such an assumption may be correct if the isothermal and adiabatic compressibility does not change during optical clearing; this is unclear due to their dependency on the longitudinal elastic modulus which is not guaranteed to remain constant during optical clearing. If the Rayleigh peak does increase after optical clearing, the performance of VIPA spectrometers that do not incorporate elastic scattering suppression can be affected [48]. 

Additionally, we reported a reduction in the linewidth of the Brillouin peaks. As mentioned in Section 2.3, the width of the Stokes Brillouin peak is related to the hyper-acoustic attenuation within the sample, to its viscoelastic properties, and even to its condensation state [49]. As also mentioned, the linewidth of a Brillouin spectrum depends on external factors as well. For instance, in the case of micro-Brillouin spectroscopy, the entrance cone of the lens not only collects backscattering but also waves that are scattered at different angles, leading to a broadening of the Brillouin peaks [50]. Additionally, microscopes with large point spread functions can lead to an overlapping of the Brillouin shifts that are produced in the irradiated volume of the sample that generates a net spectrum of large linewidth and several peaks [50,51]. We hypothesize that the reduction in linewidth upon tissue clearing is caused by an alteration of the hyper-acoustic attenuation properties of the biological tissue induced by a CUBIC-mediated density homogenization of the sample. CUBIC performs refractive index homogenization by using a high RI solution that reduces refraction between cellular and intracellular interfaces which may match the acoustic impedances by density homogenization of the sample [52]. Using the same principles as in ultrasound imaging, reflection and scattering of the acoustic waves become consequently reduced, decreasing the attenuation suffered by phonons and narrowing the FWHM of the Brillouin signal [53]. 

Some issues to be considered when combining optical clearing with Brillouin spectroscopy to reduce undesired scattering include the following:

1. Several clearing methods have high osmotic power and change the volume of the sample [52]. According to Scarcelli et al., sucrose-induced hyperosmotic shock shrinkage causes a linear increase in the Brillouin shift in NHC 3T3 cells [54]. This is a critical issue to be considered for any clearing method used in a Brillouin spectroscopic system. For instance, organic solvent-based methods such as 3DISCO or BABB or simple immersion RI matching agents such as TDE cause significant sample shrinkage during the dehydration step that may substantially alter the Brillouin frequency shift [55]. Although not reported for cellular expansion, hyperhydration clearing methods may cause similar artifacts as well.
2. The basic premise when using optical clearing to improve the Brillouin signal is that the frequency shift must not be altered. Whether this is true is not yet clear. Some experiments revealed significant differences in Brillouin shifts after decalcification with EDTA [46,47]. Additionally, it is expected that delipidating clearing agents affect the elasticity of the sample due to lipid removal [32]. Further studies of different clearing protocols are recommended.
3. Most clearing methods require 4% PFA fixation to prevent tissue degradation during optical clearing and to allow long-term preservation. PFA acts by linking lysine amino acids of contiguous protein networks and has been reported to increase the Brillouin shift of HeLa cells [49,52]. Therefore, prior fixation of optical tissue clearing methods likely hinders Brillouin signals of tissue samples.

## 3. Biological Applications of Brillouin 

### 3.1. Cell Biology Research 

Cellular stiffening is the underlying mechanism that triggers the onset of several diseases such as glaucoma, atherosclerosis, or cancer [56]. Understanding the mechanical behavior of cells could be an essential step in the characterization of such conditions. However, the evaluation of cells’ mechanical properties has been limited to artificial cellular environments that do not provide real information on in situ biological behavior. Some techniques such as AFM or magnetic bead twisting rely on applying mechanical stress to the sample, limiting their use to 2D substrates or very rudimentary 3D structures and to the cellular surface only [54]. This technique may also invoke cellular responses and alter the results [56]. Other methods such as micropipette aspiration, deformability cytometry, or optical tweezers perform mechanical assays of cells in suspension. In addition, they are invasive techniques that rely on pressure gradients to measure cellular deformability [56]. Traditional non-invasive elastography techniques such as ultrasound or MRI are limited by their low spatial resolution [56]. Studies of cells embedded in 3D hydrogels have been conducted with particle tracking microrheology but lack subcellular resolution [54]. 

#### 3.1.1. Subcellular Analysis 

Spontaneous Brillouin spectroscopy was first used for in vivo subcellular analysis in 2008 [57]. However, it was not until 2015 that it was applied to image an animal cell with subcellular resolution by coupling with confocal microscopy [54]. Prior studies were limited to the analysis of cellular components in a solution. For instance, lysozyme compressibility was quantified and protein motion and relaxation were addressed in depth [31,58]. Several studies focused on DNA revealed that phonon damping times of DNA samples depend on their hydration level due to the coupling to water relaxation [59,60]. Phonon relaxation is anisotropic, becoming stronger along the helical axis [59,61]. Additionally, the elasticity of viruses was evaluated [62]. 

**Figure 5:**
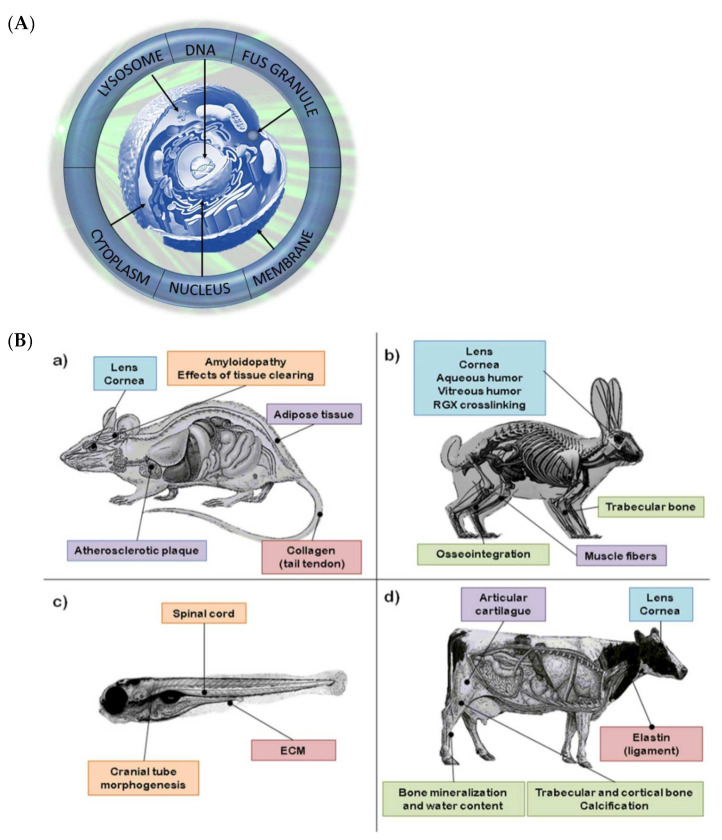

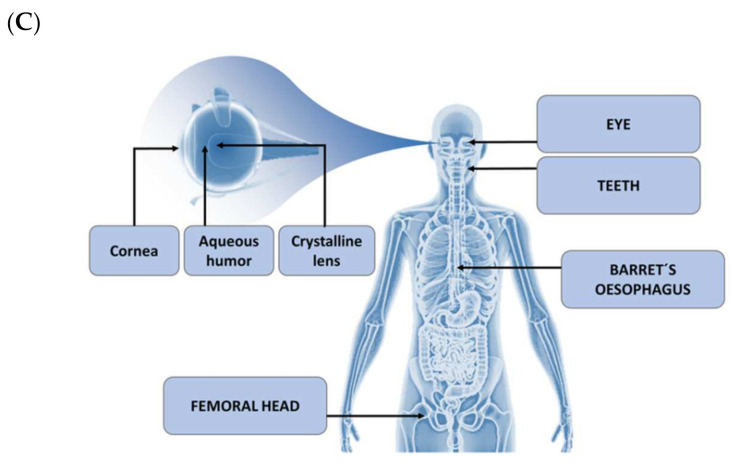
Graphical summary of all the Brillouin studies that have been performed so far regarding each of the cellular components: (**A**) animal models and (**B**) human organs or (**C**) tissues. In (**B**), the most relevant applications of Brillouin spectroscopy are organized by the animal model: (**a**) mouse, (**b**) rabbit, (**c**) zebrafish, and (**d**) cow. Many other studies have employed samples from a wide variety of animals including a pig, rat, and chicken, and less common specimens such as a trout rib, mussel, fowl, and frog.

As of 2015, BS has proved to be a valuable tool to assess the elastic behavior of cellular organelles and to image their mechanical properties (Figure 5A). Several studies have quantified the longitudinal elastic moduli of the cellular nucleus, cytoplasm, and membrane, demonstrating that the cytoplasm is softer than the rest of the cell [54,56,63]. A longitudinal elastic modulus gradient of up to 20% along the cell’s central axis has been reported [50]. Additionally, it has been shown that the environment of a cell affects its mechanical stiffness [54]. In fact, rigid environments induce cellular stiffening especially in 3D collagen ExtraCellular Matrices (ECM) compared to 2D substrates. The cellular spread area is highly correlated to such stiffening independently of the dimensionality of the environment. Cellular stiffening is also highly related to cytoskeletal-actin polymerization. By halting actin polymerization with Cytochalasin D or latrunculin-A, the cytoplasmic Brillouin shift was reported to decrease by 3.6% due to a reduction in its longitudinal modulus [54,56]. However, actin polymerization may not only affect the Brillouin shift by an induced mechanical change but also by an alteration of the refractive index of the cytoplasm as expressed by the Lorentz–Lorenz equation [64,65]:

(12) $$Rp=\sqrt{\left(2*\frac{Rm}{V}+1\right)/\left(1-\frac{Rm}{V}\right)}$$

where *R_p_* corresponds to the polymer’s refractive index, *R_m_* corresponds to the monomer’s refractive index, and *V* corresponds to the molar volume of the monomer in both the polymerized or depolymerized state. According to this expression, an increased molar volume results in a lower polymer refractive index that also accounts for a decreased Brillouin shift independent of the sample’s phonon frequency and it is known that synthetic polymers increase their molar volume upon depolymerization [22,49,66]. Natural polymers such as actin have been reported to decrease their volume upon polymerization as well, although such results are not yet conclusive [67]. In any case, reports stating that actin depolymerization affects cytoplasmic longitudinal modulus agree with posterior studies regarding which cellular stiffness is highly correlated to structural protein content or in the case of red blood cells, to functional protein concentration such as hemoglobin [50,68]. Actin depolymerization has also been shown to decrease the nuclear longitudinal modulus by 1.1% for the nucleoli and by 2.01% for the nucleus [56,69]. These results will help to better understand how nuclear stiffness is regulated and provide deeper insight into the dynamics of cell migration which is constrained by the rigidity of the cellular nucleus [69].

In 2018, BS was used to evaluate abnormal FUS protein-mediated stress granule formation which may be an underlying cause of amyotrophic lateral sclerosis (ALS) [49]. Stress granules form under stress conditions as a protection mechanism to store mRNA and dissolve in resting conditions. However, in ALS, these granules become stiffer and do not dissolve, leading to aggregations that may cause the disease. This study was conducted by using a background deflection Brillouin (BDB) confocal microscope that allowed for larger spectral resolution than traditional VIPA spectrometer-based setups [49]. Under oxidative stress, mutant FUS HeLa cells developed stress granules stiffer than the cell’s nucleus due to a liquid-to-solid phase transition, suggesting that granule stabilization may be an important cause of neurodegenerative diseases. 

There are few studies regarding cellular oncology. In 2018, BS demonstrated that induced tumoral fibroblasts have a decreased nuclear elastic modulus which is in agreement with the increased capacity of cancer cells to squeeze through narrow passages and migrate [50]. 

A NIH/3T3 cell line was used to determine the optimal laser wavelength to reduce cellular photodamage and improve Brillouin sound-to-noise ratio (SNR) [70]. Contrary to fluorescence, Brillouin scattering does not depend on light absorption but rather light absorption limits the maximum laser power that can be applied to a biological sample without causing photodamage and therefore limits the maximum achievable SNR. Nikolic et al. found that a 660 nm wavelength (*λ*) laser is 80 times less absorbed than the conventional 532 nm lasers, allowing the use of higher laser powers without inducing photodamage. Although the Brillouin signal intensity is proportional to $\lambda^{-4}$, using less energetic lasers provides for more than 30-fold SNR. 

BS has been combined with other types of imaging and spectroscopic techniques to provide a further and complete analysis of cellular samples. In 2017, spontaneous BS was coupled with flow cytometry to determine the effect of relevant physiological changes on nuclear stiffness [71]. Using VIPA spectrometers, fast sampling times, and submicron laser beams, NIH/3T3 fluorescent cells flowing through a microchannel were individually analyzed by BS and spatially co-registered by a fluorescence microscope incorporated into the setup [63]. Acquisitions were fast enough to measure four or five points within each cell, demonstrating that the nucleus is stiffer than the cytoplasm as previously reported. Trichostatin A (TSA)-induced chromatin decondensation softens the nucleus but does not affect the cytoplasm [63]. Those results agree with previous reports that conclude that nuclear stiffness does not depend on DNA content but seemingly on its condensation state [50]. 

#### 3.1.2. Microbial Biofilms 

As of 2017, several studies have used correlative Brillouin–Raman microspectroscopy to image bacterial and yeast biofilms [22,72]. They provide an insight into the composition and mechanical behavior of these highly resistant bacterial colonies. A microbial biofilm is a thick layer constituted of many prokaryotic organisms that combine to form a colony. These slim layers present interesting mechano–elastic properties due to their structure that consists of many porous layers with channels that allow the cells in the center of the colony to interchange nutrients and secrete debris. 

In the case of Scarponi et al., this Brillouin–Raman coupling was possible due to the use of a tandem Fabry–Perot interferometer combined with optical insulators, allowing Brillouin acquisition times to better fit those of Raman but without losing spectral resolution nor contrast. By simultaneous detection of both scattering phenomena, it was found that the center of the biofilm had increased the Raman signal due to the presence of cytochrome c [22,73]. A decreased Brillouin shift was caused by higher hydration levels [22,72], demonstrating higher cell viability at the core of the biofilm. Contrarily, other Brillouin–Raman studies have shown that different types of biofilm present increased stiffness in the center; notably, mostly small colonies due to a growth model characterized by an outer edge proliferation [74]. 

### 3.2. Animal Tissue Biology: Preclinical Studies 

Brillouin light scattering techniques for assessing tissue stiffness in animals have covered an extensive range of animal samples and disease models. They have offered new insight regarding the role of tissue stiffness in a variety of biological processes, demonstrating the adequacy and sensitivity of BS for many physiological processes and paving the way for translational and clinical applications (Figure 5B). 

#### 3.2.1. Vascular Applications 

Brillouin microscopy was validated in 2015 as a method to measure the stiffness in atherosclerotic vessel sections as it is the only factor that influences plaque stress (local stiffness, geometry, and blood pressure) that cannot be measured with the technologies currently available in clinics. The study demonstrated a direct relationship between frequency shift and collagen presence, and observed a strong inverse correlation between the Brillouin frequency shift and the local accumulation of lipid in an atherosclerotic mouse artery. The group further proposed a combined optical coherence tomography and BS catheter system to obtain information regarding both stiffness and geometry in vivo simultaneously [75]. 

#### 3.2.2. Nervous System Applications 

##### CSF Screening 

BS has also shown the ability to detect the increase in protein concentration in cerebrospinal fluid (CSF) that is associated with bacterial meningitis disorders [76]. Early diagnosis is vital to reduce this condition’s lethality and prevent the physician from performing further tests. Standard methods require many reagents that are not sensitive enough or partially destroy the sample. BS has detected abnormally high protein concentrations in CSF that is associated with most bacterial meningitis cases with a high level of confidence in a study using model fluids emulating healthy and diseased CSF composition. When coupling these results to the observation of common symptomatology, BS demonstrates a bright future as a diagnostic tool for meningococcal inflammatory diseases that could significantly accelerate the diagnostic process [76]. 

##### Amyloid Plaques in Alzheimer’s Disease 

Amyloidopathy is one of the most common hallmarks of Alzheimer’s disease. It is characterized by deposits of the amyloid-β polypeptide within the parenchyma of the brain. It has been regarded for the last three decades as the leading underlying cause of Alzheimer’s disease. Moreover, it is related to the deterioration of cognitive and neurophysiological function. Correlative Brillouin–Raman spectroscopy was used in 2017 to map β-amyloid plaques’ stiffness in the hippocampus in a transgenic mouse model. Detailed viscoelastic characterization of amyloid plaques was reported using Brillouin to map the stiffness and micro-Raman to give the molecular structure and composition [77]. A different study in 2018 used a method based on non-negative factorization (NMF) to apply a multivariate statistical model to decompose the hyperspectral dataset in distinct components, allowing to distinguish between four different plaque constituents [78]. 

#### 3.2.3. Ophthalmological Applications 

BS and microscopy have been widely applied to the cornea and the crystalline or lens. The first application of BS for the study of the eye was in 1980, determining the refractive index in different ocular tissues including the cornea, capsule, and lens, and estimating the real and imaginary parts of the longitudinal elastic modulus as determined by BS [11]. Values of density and hypersonic speed were also obtained for various species including the human cornea. The study revealed that the protein content density and refractive index of the lens increases radially towards the center. Vaughan and Randall further extended their analysis to fifteen species of animals in a different study from 1982 in which they focused solely on the lens. In this case, they measured values of speed and hypersonic attenuation, finding significant differences between animals and different areas of the same lens [79]. 

In the last two decades, the possibilities regarding the use of Brillouin microscopy on both the lens and cornea have gained great interest and resulted in significant advances in pushing Brillouin microscopy-based technologies closer to the clinic. 

##### Cornea 

Brillouin has been proved to be a feasible tool for the early diagnosis and monitoring of keratoconus and corneal ectasia following LASIK (laser-assisted in situ keratomileusis) surgery [80]. Both conditions involve the loss of rigidity and thinning of the cornea, leading to corneal protrusion, and can be treated by increasing corneal stiffness through riboflavin collagen photo-crosslinking (CXL). The depth-dependent longitudinal modulus in the bovine eye before and after CXL treatment was 3D imaged, validating Brillouin imaging to measure the crosslinking degree of the cornea in a contactless manner [80,81]. BS was also used in the evaluation of corneal collagen photo-crosslinking by rose bengal and green light (RGX) in rabbit eyes [82]. 

Further studies of the cornea’s biomechanics and biophysics can explore issues regarding, for example, how to improve cornea tissue bonding following cataract surgery. 

##### Lens 

Regarding the crystalline*,* in situ biomechanical measurements in mice along the optical axis, with the eye remaining in place, were reported [35]. Reiβ et al. measured the longitudinal modulus (M) for a rabbit, pig, and a 70-year-old human eye in an axial manner, proving the aqueous humor, vitreous humor, and lens to be distinguishable [83]. They later revealed that postmortem changes in the Brillouin shift of in vitro porcine lenses were observed in as soon as 3 h after death [84]. 

#### 3.2.4. The ECM and Its Fibrous Proteins 

The extracellular matrix (ECM) provides structural scaffolding to cellular constituents. It initiates cues that trigger and regulate various biochemical pathways, influencing morphogenesis, homeostasis, and cellular differentiation processes [85]. Stiffness of the ECM is primarily dependent on collagen and elastin concentrations and has been shown to have a significant role in regulating cell function [86]. Several studies have used Brillouin light scattering to characterize a range of fibrous ECM proteins and study their mechanical behavior. 

The first application of BS to the study of ECM proteins took place in 1977 in which the Brillouin shift for type-I collagen from a rat tail tendon was reported. This was the first application of BS to biopolymers and it was noted that drying the sample increased the stiffness [10]. Later on, these results included measurements of the anisotropic nature of elastic properties in collagen [87]. Assuming dry collagen fibers behave in a transversely isotropic manner, following a cylindrical symmetry model, they characterized them in terms of the five independent elastic constants corresponding to such a system. Results for wet collagen were incomplete. In both studies, the elastic moduli values were significantly higher than those determined by quasistatic methods at the macroscopic scale, suggesting a viscoelastic behavior. A different study published that same year reported the Brillouin shift for wet unstretched rat tail collagen, horsehair keratin (stretched and unstretched), and two synthetic polypeptides in thin films known to have an αhelix conformation [88]. Observations on keratin suggested that Brillouin frequency shifts may be related to the fiber’s water content (see Section 2.3) and the degree of helical extension. 

Following these contributions, there was a lapse of thirty years. Further examination of ECM proteins using BS was not pursued except for an additional report of wet rat tail axial velocity in 1990 [89]. Palombo et al. resumed this line of work in 2014, reporting new measurements on type I and II collagen from a rat tail tendon, articular cartilage, and ligament elastin [90]. Adopting the cylindrical symmetry model established by Cusak and Miller, they offered the first full characterization of elastin, cartilage, and ligament fibers in terms of their elastic tensors. Again, the fibers examined were found to behave in a viscoelastic manner with the elastic moduli depending on the probing frequency. 

A protocol for extraction and preparation of trypsin-digested type-I collagen fibers and elastin was presented in 2016, focusing on the fibers’ frequency dependency once again [91]. Finally, an additional study tested the mechanics of purified collagen and elastic fibers for the influence of fibrillar structure and hydration level, obtaining a full characterization of the mechanical tensor and elastic module [92]. Compared with data from the quasistatic measurements at different hydration levels, these new results provided the full description of fiber viscoelastic behavior. 

Following a different line of research, an in vivo Brillouin imaging of zebrafish ECM at a high resolution and in 3D was reported to gain insight into its influence in the morphogenesis of the embryonic notochord whose cylindrical shape is influenced by the ECM mechanical integrity [93]. Further investigation in this field could be extended to provide new information regarding the elongation processes and branching of tubular structures that divide into structures such as mammary ducts or renal tubules. 

#### 3.2.5. Other Applications in Animal Models 

##### Melanoma 

Recent studies by Troyanova-Wood et al. evaluated Brillouin micro-spectroscopy in Sinclair miniature swine as a potent tool to distinguish between healthy skin, regressing melanoma, and non-regressing melanoma. Measurements of the Brillouin shift between the three types of samples were statistically significantly different, with non-regressing melanoma observed as stiffer than regressing melanoma and healthy tissue observed as the softest of the three. The results suggest that BS holds valuable potential as a diagnostic and monitoring tool in treating melanoma that would enable physicians to find tumor boundaries with high accuracy [94,95]. 

##### Muscle 

Not much experimental work involving Brillouin microscopy has revolved around the study of muscle tissue. The first report of the Brillouin frequency shift measurement of muscle was in the dry anterior byssus retractor, measuring rat tail tendon collagen [10]. A decade later in 1989, muscle tissue was investigated using BS to obtain the Brillouin shift from relaxed, glycerinated single muscle fibers from a rabbit psoas muscle ex vivo [96]. Our group offered measurements of the frequency shift and hypersonic attenuation from mice myocardium in a publication in which the impact of tissue clearing on Brillouin studies was explored [45]. 

##### Adipose Tissue 

Obesity is associated with changes in the chemical and mechanical structure of tissues. A study published in 2017 used correlative Brillouin–Raman spectroscopy to evaluate these variations in the white and brown adipose tissue of diet-induced obese mice. Results indicated that adipose tissue experiences an increase in stiffness and lipid content as a result of obesity. These changes were more noticeable in the brown adipose tissue [97]. 

##### Bone and Articular Cartilage 

The consensus reached by the National Institutes of Health in 2000 stating that bone quality and bone mineral density (BMD) should be considered when diagnosing osteoporosis has emphasized the need for the development of new tools capable of assessing traits such as mineralization or damage accumulation arose (Online, 2000). In the last decades, several studies have evaluated both the properties of bone in the GHz range and the potential of BS in the analysis of bone quality, both on its own and in conjunction with traditional bone characterization techniques such as nanoindentation and radiography, and the first Brillouin measurements of bone corresponded to a wet mineralized trout rib bone [87]. A decade later, the different mineralized compact tissues comprising measurements from a turkey leg tendon, deer antler, and cow tibia were described. Values for the hypersonic velocities in wet, dry, mineralized, and dematerialized samples were obtained [89]. The same study also reported new measurements for rat tail collagen. A more recent study measured the wave velocity in a bovine femoral cortical bone and compared it to a dry collagen film. The measured wave velocity was much higher than the one provided by ultrasonic wave methods that operate in the MHz range [98]. In 2011, micro-Brillouin spectroscopy was used to evaluate the differences in the mechanical properties of mature and newly formed bone. The integrated response of rabbit bone was studied in vivo after implantation of titanium pieces, validating the technique as a potential tool for studies concerning implant osseous integration [99]. Micro-Brillouin was used to characterize bones’ mechanical properties to report hypersonic velocities from thin, translucent specimens from the bovine and cortical trabecular bone [47]. 

In parallel, a study compared measurements of the mechanical properties of a trabecula obtained from micro-Brillouin spectroscopy with those obtained from SAM. The data suggested that micro-Brillouin was very useful for the study of trabecular anisotropy in correlative studies with traditional techniques due to its ability to obtain measurements in various directions for a given specimen as opposed to SAM or nanoindentation [100]. Trabecular anisotropy has also been studied via Brillouin light scattering. Their results demonstrated that bone hypersonic wave properties are likely to change with a trabecular structure [101]. 

Regarding bone calcification’s impact on its mechanical properties, Fukui et al. have used a micro-Brillouin technique to obtain the hypersonic wave velocity in the cortical bone from a cow femur. They estimated its relation with hydroxyapatite content and demonstrated that decalcification caused a significant wave velocity decrease, thus reducing stiffness [46]. 

Finally, a recent study evaluated the variations in Brillouin shift from bones subjected to compression loads in chicken, sheep, cow tibia, and rabbit ulna, finding that Brillouin shifts were higher for successively increasing loads, followed by a slight decrease. The efficacy of several tissue engineering approaches was also studied in vivo for critical-sized defects, focusing on heparin-conjugated fibrin (HCF) hydrogels, bone morphogenic proteins, and both osteogenic and mesenchymal stem for bone regeneration in rabbit ulna. Moreover, the study also reported that bones with fully consolidated fractures have higher elastic moduli than bones with defects [102]. 

Wave velocities for dry and wet articular cartilage from a bovine femur were obtained by BS, demonstrating that the cartilage closer to subchondral bone was stiffer. Weak anisotropy and a dependency on water content were also found [103]. 

### 3.3. Brillouin in Development Biology 

#### 3.3.1. Zebrafish Spinal Cord 

Injury to the spinal cord leads to signaling pathways, resulting in a fibrotic scar that have been shown to prevent axonal growth and regeneration across the lesion site. The first application of confocal Brillouin microscopy to zebrafish larvae’s spinal cord tissue was reported, analyzing its mechanical properties during development and following spinal cord lesions [104]. Results demonstrated that pathological and developmental processes matched noticeable variations in Brillouin shifts, and the longitudinal modulus (elasticity) and viscosity of distinct larval tissues were explicitly obtained. Furthermore, differences were found between in vivo and ex vivo measurements. The contactless and non-invasive nature of Brillouin microscopy renders it as a potent tool for identifying and evaluating the relative importance of tissue mechanics in developmental and regenerative processes involving biochemical and genetic factors. 

#### 3.3.2. Mouse Embryogenesis 

Mechanical stimuli significantly impact organogenesis and morphogenesis. The most relevant mechanical factors are the spatial distribution of stresses generated by the cells and the local environment properties. The Brillouin shift of full murine embryos was imaged by BS with the aid of optical coherence tomography for structural guidance in a pilot study [105]. A different study used the same technology to map the longitudinal modulus of mouse embryos, revealing that Brillouin-OCT was sensitive enough to characterize the changes in mechanical properties in the neural tube region during embryogenesis [106]. 

#### 3.3.3. Pharynx of Caenorhabditis elegans 

Stimulated Brillouin scattering has been recently applied to *Caenorhabditis elegans* which are a semi-transparent nematode that inhabits warm soil environments to quantify the changing stiffness of the pharynx that is known to increase upon hyperosmotic shock [20]. 

### 3.4. Applications of Brillouin to Diagnosis 

Efforts are being made to develop clinically viable devices that allow physicians to take advantage of BS’s unique properties as a potential pathology diagnostic tool. Up to the present, Brillouin studies of human organs are scarce and have been mainly focused on ocular research in which human trials for an in vivo diagnostic tool have already taken place (Figure 5C). However, results are promising and may prove BS as a novel pathology diagnostic tool. 

#### 3.4.1. Ophthalmology 

Since the first study of the human eye was performed in 1980 [11], the topic was left untouched until the crystalline lenses belonging to 19 donors of different ages were evaluated [107]. They found that the average value of the longitudinal elastic constant of the lens’ nucleus is significantly larger than that of the cortex. These results were confirmed later and it was demonstrated that the stiffness of neither the lens’ cortex nor the nucleus significantly changed with age [53]. These data suggested that presbyopia, caused by the lens’ reduced accommodation capacity, does not arise due to a stiffening of the lens as it was believed. As of then, the topic gained significant interest (Figure 1A) and several studies were published in the following decade. Posterior studies found that the lens’ nucleus thickens with age [108]. Although its longitudinal elastic modulus does not change as previously reported by Bailey, S. T. et al., a thickening of the nucleus may be the real underlying mechanism related to the arousal of presbyopia. Later studies reported a significant age-related change of corneal stiffness [109,110,111]. 

In recent years, other ophthalmological abnormalities have been related to a change in their elasticity. For instance, several studies performed on corneas suffering from keratoconus revealed an alteration of the corneal elastic properties, limited to the cone region, suggesting that corneal deformation is caused by an increase in its elasticity [109,111,112]. This leads to a reduced capacity to withstand the intraocular pressure, perhaps caused by the loss of collagenous sutural lamellae [112,113]. Interestingly, it was found that such variation in elasticity is asymmetric between both eyes [110]. 

Healthy corneas are stiffer in their center and softer in their periphery [48,114]. This mechanical gradient plays a major role in the proliferation and differentiation of limbal epithelial stem cells (LESC) that mainly reside in the soft periphery of the cornea. LESC are fundamental for the proper preservation of the cornea and can be used as a potential therapy for damaged corneas by regulating the stiffness of the corneal substrate [114].

It has also been found that healthy corneas become softer during sleep due to the decreased evaporation through closed eyelids and the consequent increase in corneal hydration [115]. 

#### 3.4.2. Bone 

Human bone has not been thoroughly analyzed by BS yet. Up to the present, one of the few studies that have been performed examined small volumes of the femoral head. Two pairs of frequency peaks were detected for each of the volumes’ layers; that is, the articular cartilage, subchondral bone, and trabecular bone [116]. The low-frequency shift peak (i.e., the smallest Brillouin shift) had never been reported before and was attributed to a soft tissue component caused by thin and poorly organized collagen fibers. In contrast, the high frequency peak was assigned to the mineralized and ordered collagen meshes within each layer [116].

## 4. Brillouin as a Pathology Diagnosis Tool 

BS has a promising future as a clinical diagnostic tool in ophthalmology [117] and has already been implemented into clinically viable instrumentation systems [44,110,112]. It has been used to obtain an in vivo axial profile of the longitudinal elastic modulus of a patient’s eye by sequentially focusing the laser beam at different depths and detecting the corresponding backscattering, revealing significant differences in elasticity between the cornea, the aqueous humor, and the cortex and nucleus of the crystalline lens [44]. Such analysis can help diagnose pathologies such as keratoconus, cataracts, or corneal ectasia, detecting abnormal elasticity values in addition to the depth profile before morphological changes are manifested [111,117,118]. However, current Brillouin instrumentation is neither sensitive nor specific enough for detailed disease characterization than compared to other currently available diagnosis techniques [109]; therefore, further development is required before clinical implementation. 

It has been shown that the relative angle between the probing laser beam and the cornea is fundamental in the interpretation of the Brillouin data [119]. Due to the anisotropy of the fibrillar collagen mesh of the cornea, different measurements can be obtained depending on the relative angle, similarly to what is shown in Figure 4. Such dependency could be exploited in the diagnosis of corneal degenerative diseases in which the structural anisotropy is lost.

Several parameters must be adapted to compatibilize BS with ophthalmological diagnosis and avoid harming the patient. For instance, laser beams of larger wavelengths than the conventional 532 nm must be used to diminish tissue absorption and their power must be reduced below the maximum permissible exposure (MPE) to avoid thermal biohazards [44,70]. For the same reason, the beam width must be large enough to prevent excessive power concentration at unfocused regions. The scan must be performed quickly to reduce retinal exposure and avoid movement artifacts and patient discomfort [44]. However, this indicates that the Brillouin signal will present a low intensity due to the laser’s soft power, fast acquisition times, and considering the large wavelength used as Brillouin intensity is inversely proportional to the fourth power of laser wavelength [70]. Therefore, the ideal acquisition system would combine the advantages of a VIPA spectrometer which is characterized by its fast spectral decomposition and a Fabry–Perot interferometer that provides excellent signal resolution and contrast [80]. However, according to Scarcelli et al., a two-stage VIPA spectrometer seems to provide good enough resolution likely due to the transparency and low turbidity of the eye [44,80]. 

Additionally, BS could be coupled to an in vivo arthroscopy diagnosis system to evaluate the loss of extracellular matrix proteoglycan. Such a loss leads to a change in articular cartilage’s water content which is one of the possible causes that can lead to osteoarthritis (OA) [33]. For highly hydrated tissues such as the articular cartilage, the Brillouin shift is very sensitive to water content as discussed in Section 2.3. In the cornea, a hydration increase correlated to a decrease in frequency shift [115]. The dependence of the Brillouin frequency shift on the sample’s water content establishes Brillouin microscopy as an order of magnitude more sensitive than MRI or CT in the detection of OA and classifies it as an excellent clinical tool for the detection of corneal endothelial diseases [33,115]. Therefore, in the future, BS could be used to optimize the early-stage diagnosis of several hydrations-related disorders. 

Further applications of BS as a clinical tool include melanoma diagnosis by comparison with local elasticity of surrounding healthy tissue to improve tumor margin assessment or monitor tumor progression [94,95]. Additionally, BS holds the potential to aid decision-making in dentistry by helping to accurately delineate carious dentin [120].

Finally, a combination of Brillouin mechanical mapping with Raman chemical fingerprint analysis can provide valuable histological information for ex vivo diagnoses of biopsies. For instance, mechanical and chemical maps of Barrett’s esophagus epithelial metaplasia have been imaged [121]. Brillouin–Raman spectroscopy has been used as a high-resolution histological tool to detect neoplastic tissue changes at a cellular level by combining it with multivariate statistical analysis such as principal component and k-means clustering [122]. If future technological advances allow similar coupling of Brillouin and Raman spectroscopies at smaller scales, a similar analysis could become available for in vivo diagnosis. This advance should be possible using miniature endoscopic probes that are currently available for Raman and Brilluoin independently or in situ evaluation of biopsies by Brillouin–Raman coupled hypodermic needles for assessment of critical injection sites [39,40,123,124,125]. 

As a final note, Brillouin–Raman spectroscopy can also become a valuable tool to determine skin health status and diagnose possible injuries by analyzing its viscoelastic properties or to assess the intensity of burn injuries [126,127]. However, most studies regarding skin have been performed on animal models only. 

## 5. Conclusions 

Brillouin imaging has emerged as a promising tool for characterizing biological samples in terms of their viscoelastic behavior. Subcellular components, cells, animal tissues, and human samples have been analyzed by this technique. BS holds valuable potential to identify regressing and non-regressing melanoma, carious dentin, CSF bacterial meningitis, and atherosclerotic vessels, among other conditions. It can become a very valuable diagnostic and monitoring tool in the treatment of melanoma, ophthalmological diseases, osteoarthritis, burn injuries, and many other disorders, enabling physicians to quantitatively assess their patients’ status. 

Considering these advances, BS is regarded as a new generation diagnostic tool.
